# Met@MPDA rejuvenates BMSC energy metabolism to promote bone regeneration in semaglutide-treated obese periodontitis

**DOI:** 10.1016/j.mtbio.2026.103231

**Published:** 2026-05-14

**Authors:** Ting Jiang, Tian-Hao Wan, Min-Jie Wang, Xue-Qin Zhu, Yu-Ran Jiang, Feng Yang, Zhi-Chen Ling, Xin-Yi Tan, Jun Wang, Ning-Juan Ouyang

**Affiliations:** a Department of Pediatric Dentistry, Shanghai Ninth People's Hospital, Shanghai Jiao Tong University School of Medicine, China; b Department of Oral Surgery, Shanghai Ninth People's Hospital, Shanghai Jiao Tong University School of Medicine, China; c Department of Orthodontics, Shanghai Ninth People's Hospital, Shanghai Jiao Tong University School of Medicine, China; d College of Stomatology, Shanghai Jiao Tong University, China; e National Center for Stomatology, National Clinical Research Center for Oral Diseases, China; f Shanghai Key Laboratory of Stomatology, China; g Shanghai Research Institute of Stomatology, Research Unit of Oral and Maxillofacial Regenerative Medicine, Chinese Academy of Medical Sciences, China; h Oral Bioengineering Lab, Shanghai Key Laboratory of Stomatology & Shanghai Research Institute of Stomatology, China; i National Clinical Research Center of Stomatology, Shanghai, China

**Keywords:** Obesity, Periodontitis, Semaglutide, FAO, AMPK

## Abstract

Obesity is closely linked to periodontitis development. Although semaglutide is increasingly used to manage obesity, its effects on alveolar bone repair in periodontitis are not well understood. In a mouse model of obesity-related periodontitis, we found that obesity exacerbates alveolar bone loss in an inflammatory setting. Semaglutide treatment slightly reduced periodontal inflammation and osteoclast markers but did not improve osteogenesis or regenerate alveolar defects. Transcriptomic analysis revealed increased fatty-acid uptake but suppressed AMPK signaling and reduced fatty-acid oxidation (FAO) in inflamed alveolar bone of obese mice. *In vitro*, macrophage FAO was largely unaffected by inflammation or semaglutide, while FAO in bone marrow–derived mesenchymal stem cells (BMSCs) was significantly reduced, impairing osteogenic differentiation. To tackle metabolic and osteogenic issues, we created a metformin-loaded mesoporous polydopamine system (Met@MPDA) that acts as an AMPK agonist. Met@MPDA is absorbed by BMSCs, restoring cellular energy and promoting alveolar bone regeneration in periodontitis. While semaglutide provides anti-inflammatory benefits in obesity-related periodontitis, it falls short in boosting bone regeneration due to metabolic issues in BMSCs. Thus, Met@MPDA's sustained metformin delivery could enhance bone regeneration in these cases.

## Graphical abstract

Obesity aggravates periodontitis by amplifying inflammatory burden and accelerating alveolar bone loss. In obese mice, systemic semaglutide attenuates inflammation, yet bone regeneration remains suboptimal. Met@MPDA-Gel enhances osteogenesis by rewiring BMSC energy metabolism to favor bone formation. Importantly, the combination synchronizes anti-inflammatory efficacy with anabolic bone repair, thereby mitigating periodontal destruction in the context of obesity. Collectively, these findings suggest that, alongside semaglutide-based obesity management, Met@MPDA-Gel represents a promising adjunctive therapy for obesity-associated periodontitis.

**Figure:**
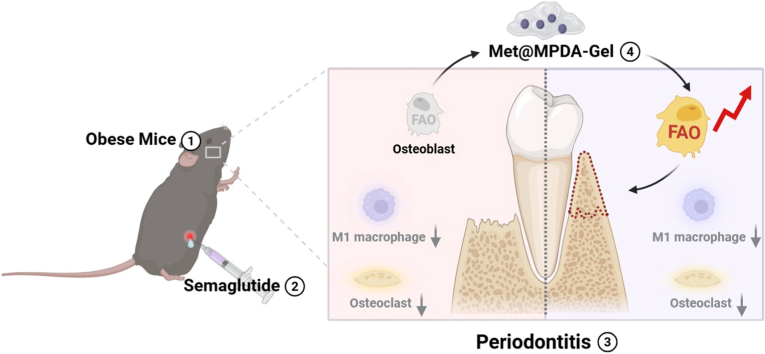

## 1 Introduction

Periodontitis is a multifactorial chronic inflammatory disease characterized by the progressive destruction of tooth-supporting tissues, ultimately leading to alveolar bone loss and tooth mobility [1]. Notably, periodontitis extends beyond being a localized oral infection; it is a prevalent chronic inflammatory condition intricately associated with a range of systemic comorbidities, such as cardiovascular disease, diabetes mellitus, and obesity [2,3]. While conventional periodontal therapies are effective in controlling inflammation, they typically result in repair rather than true regeneration, leaving significant alveolar bone defects that compromise both mastication and esthetics [4]. Consequently, the regeneration of damaged alveolar bone remains a significant clinical challenge, particularly in patients with systemic comorbidities that impair the host's healing capacity, making treatment outcomes more difficult to predict and optimize.

Over the past five decades, the prevalence of obesity has increased significantly, with rates among adults aged 18 years and older rising from 7% to 16% [5]. Obesity has emerged as a critical systemic risk factor for periodontitis, demonstrating a strong association with increased prevalence, heightened disease severity, and an elevated risk of disease progression [6]. This correlation is primarily attributed to chronic low-grade systemic inflammation and immunometabolic dysregulation associated with obesity [7,8]. In individuals with obesity, adipose tissue functions as an active endocrine organ, secreting pro-inflammatory adipokines such as TNF-α, IL-6, and leptin. These adipokines exacerbate local periodontal inflammation and create a microenvironment that is detrimental to tissue repair [9,10]. Furthermore, metabolic stress resulting from obesity can impair the regenerative capacity of endogenous progenitor cells, making obesity-associated periodontitis particularly challenging to manage [11]. This highlights the necessity for therapeutic strategies that address both local inflammation and systemic metabolic factors. The simultaneous increase in obesity and periodontitis presents an escalating challenge to global healthcare systems. Consequently, preventive measures in accordance with World Health Organization (WHO) guidelines, combined with integrated treatment frameworks that concurrently address obesity and periodontal health, have the potential to deliver significant and wide-ranging benefits for individuals and society as a whole [12,13].

Semaglutide, a glucagon-like peptide-1 receptor agonist (GLP-1RA), has significantly transformed the therapeutic landscape of obesity due to its potent weight-reducing efficacy [14]. Beyond its roles in weight management and glycemic control, emerging evidence indicates that semaglutide possesses anti-inflammatory and immunomodulatory properties and may also impact bone metabolism [15]. Experimental studies demonstrate that semaglutide reduces systemic inflammatory mediators such as TNF-α and IL-6 and mitigates oxidative stress in obese mice [16]. In models of obesity induced by a high-fat diet, preoperative administration of semaglutide further decreases the risk of implant-associated bone and joint infections [17]. Research focusing on bone health suggests that semaglutide can enhance the proliferation, migration, and osteogenic differentiation of bone-derived mesenchymal stem cells, upregulate osteogenic markers, and activate the Wnt/LRP5/β-catenin pathway [18]. Notably, disruption of Wnt signaling partially diminishes these pro-osteogenic effects [19]. Simultaneously, GLP-1 signaling has been documented to inhibit osteoclast differentiation and suppress bone resorption, thereby potentially aiding in the preservation of bone mass in osteoporotic models [18]. However, within the framework of obesity-associated periodontitis—characterized by increased inflammation and disrupted bone metabolism—the precise effects of semaglutide on inflammatory alveolar bone loss and its subsequent repair remain inadequately understood.

Bone remodeling is a meticulously regulated process orchestrated by osteoclasts, osteoblasts, and osteocytes, and is contingent upon the dynamic coupling and homeostatic equilibrium between bone resorption and bone formation. In the context of periodontitis, chronic inflammation perturbs this equilibrium: a local environment enriched with pro-inflammatory mediators, such as receptor activator of nuclear factor kappa-Β ligand (RANKL), promotes osteoclastogenesis and activation, resulting in progressive alveolar bone loss. Concurrently, inflammation may compromise the osteogenic differentiation potential of bone marrow mesenchymal stem cells (BMSCs), a phenomenon often exacerbated by obesity and other metabolic disorders [20]. Macrophages serve as a critical nexus, linking the regulation of inflammation to bone regeneration. The polarization of macrophages significantly influences the inflammatory trajectory and determines whether a pro-inflammatory or pro-regenerative microenvironment is established. Specifically, M1 macrophages are linked to inflammation and tissue damage, while M2 macrophages facilitate the resolution of inflammation and promote tissue repair. Therefore, successful bone regeneration necessitates an initial thorough evaluation and modulation of the inflammatory microenvironment. This should be followed by the implementation of antiresorptive interventions alongside strategies aimed at restoring and enhancing the osteogenic potential of BMSCs, ultimately re-establishing the balance of bone remodeling.

Cellular energy metabolism plays a crucial role in determining cell phenotype and function [21]. Recent advancements in the field of immunometabolism have elucidated that various cell types employ specialized metabolic pathways to fulfill their specific biological requirements. For instance, macrophages primarily utilize glycolysis during episodes of acute inflammation but transition to fatty acid oxidation (FAO) during the resolution of inflammation and tissue repair processes [22]. Similarly, BMSCs encounter significant bioenergetic demands during osteogenic differentiation, with growing evidence underscoring the critical role of FAO in supporting osteogenesis [23]. Nevertheless, under adverse conditions such as chronic inflammation or obesity, metabolic flexibility is often compromised, which may limit the osteogenic potential of BMSCs. Adenosine monophosphate-activated protein kinase (AMPK) serves as a cellular energy sensor and a key regulator of metabolic homeostasis. Activation of AMPK facilitates FAO, enhances mitochondrial biogenesis, and suppresses pro-inflammatory signaling pathways. Consequently, the AMPK–FAO axis constitutes a promising and targetable pathway for therapeutic intervention. Reactivation of AMPK activity has the potential to “re-energize” BMSCs while simultaneously reducing local inflammation. This approach offers a mechanistically substantiated strategy to address the regenerative limitations observed in obesity-associated periodontitis.

Metformin is a well-recognized agonist of AMPK, with an established profile of safety and efficacy. As a commonly prescribed antidiabetic medication, its effects on bone health have garnered increasing scientific interest. Research indicates that metformin facilitates the osteogenic differentiation of dental pulp stem cells via AMPK signaling pathways and can restore the osteogenic potential of adipose-derived stem cells impaired by hyperglycemic conditions through the activation of autophagy [24,25]. Furthermore, metformin demonstrates significant anti-resorptive properties by inhibiting osteoclastogenesis and osteoclast activity, thereby mitigating bone resorption [26]. Its potential application in bone tissue engineering is also noteworthy; for instance, the incorporation of metformin into nanofibrous scaffolds has been shown to significantly enhance the healing of bone defects [27]. Additionally, mesoporous polydopamine (MPDA) nanoparticles have emerged as promising drug delivery vehicles due to their superior biocompatibility, favorable photothermal properties, and reactive surface chemistry, which enable efficient drug loading. In this study, we developed a metformin-loaded MPDA sustained-release system (Met@MPDA) designed to facilitate localized delivery and prolonged release of metformin. This approach aims to optimize metabolic recovery in BMSCs while minimizing systemic exposure.

Our research suggests that obesity-related immunometabolic dysfunction impairs BMSC osteogenic differentiation, limiting alveolar bone regeneration even with semaglutide's anti-inflammatory and anti-resorptive benefits. Impaired AMPK–FAO activity and fatty acid oxidation in BMSCs hinder regeneration, indicating the need to restore BMSC energy metabolism. To address this, we developed the Met@MPDA sustained-release system to be internalized by BMSCs, activate AMPK, and enhance bone repair, offering a metabolism-focused therapy for periodontitis in obese patients.

## 2 Materials and methods

### 2.1 Mice

4-week-old male C57BL/6J mice were housed as 5 mice/cage. They received water ad libitum and were exposed to a 12 h light/dark cycle and 10% humidity at 20 ± 4 °C. The mice were adaptively fed for one week. Mice were fed a standard diet (normal control group) or high-fat diet (HFD, 60 kcal% fat, D12492) throughout the experiment. After 8 weeks of feeding, mice were used for subsequent experiments. Mice fed the HFD were classified as obese, whereas mice fed the normal diet were classified as nonobese. A modified mouse model of ligature-induced experimental periodontitis was established as previously described [28]. Semaglutide-treated mice received semaglutide (MedChemExpress, #HY-114118, 40 μg/kg, subcutaneous injection, every 2 days) [29]. Normal control mice were injected with the saline vehicle. Throughout the study, body weight levels were measured weekly.

### 2.2 Micro-CT

Mice were euthanized at the designated endpoints, and maxillary specimens were harvested and fixed in 4% paraformaldehyde. The maxillae were then scanned using micro-computed tomography (μCT 50 cabinet microCT scanner, SCANCO Medical AG, Bassersdorf, Zurich, Switzerland) and reconstructed in three-dimensional (3D) to assess alveolar bone loss. Dissected maxillae were horizontally placed into the sample tubes, and parameters were set: 55 kVp, 177 mA, and 8 μm resolution to start the scan. The 3D reconstruction was performed using Amira-Avizo software (Version 2020.1, Thermo Scientific). The reconstructed images were captured by complying with the identical criteria that all the tooth cusps were located on the same plane, and the occlusion plane could not be seen from the buccal side or the palatal side. The linear distance from the cementoenamel junction (CEJ) to the alveolar bone crest (ABC) was used as the primary outcome and measured at predefined sites on the palatal aspects of the maxillary second molars; mean values were calculated for statistical analysis. In addition, a region of interest (ROI) was selected from the reconstructed datasets for quantitative morphometric analysis, and parameters such as bone volume fraction (BV/TV) and trabecular number (Tb.N) were calculated to comprehensively evaluate periodontitis-associated alveolar bone destruction.

### 2.3 Histological analysis

The fixed samples were decalcified with 10% disodium ethylenediamine tetraacetate (EDTA) for 4 weeks. Afterward, the specimens were dehydrated in a graded series of ethanol solutions. After the transparent treatment with xylene, the specimen was embedded in paraffin to obtain paraffin sections with a thickness of 5 μm. Hematoxylin and eosin (H&E) and Trap staining was performed to assess periodontal tissue regeneration. Immunohistochemical staining for CD68 (1:500, Abcam, #ab283654), CD86 (1:500, Abcam, #ab119857) and CD206 (1:500, Proteintech, 83485-1-RR) was performed to assess the immunomodulatory effects and immunohistochemical staining for ALP (1:500, Abcam, #ab95462) and CD36 (1:500, Abcam, #ab252923) were performed to assess the osteogenic effects. The staining of specimens was observed under a microscope and the positive cells per high power field were calculated.

### 2.4 Tissue flow cytometry

Maxillary periodontal tissues were harvested, placed in ice-cold phosphate buffer solution (PBS), and minced into small fragments. The samples were enzymatically digested at 37 °C with gentle agitation in digestion buffer containing collagenase (type I/IV) and DNase I to generate single-cell suspensions. The digestion was quenched with cold complete medium containing fetal bovine serum, and the suspension was filtered through a 70-μm cell strainer. Cells were centrifuged, washed with PBS supplemented with 1–2% FBS. Cell numbers were determined, and samples were incubated with Fc receptor blocking reagent, followed by staining with a fixable viability dye. For surface marker staining (CD68, CD86), cells were incubated with fluorochrome-conjugated antibodies at 4 °C in the dark, washed, and resuspended in staining buffer. For intracellular staining (CD206), cells were fixed and permeabilized using a commercial fixation/permeabilization kit and then incubated with the indicated intracellular antibodies. Data were acquired on a flow cytometer and analyzed using *FlowJo* (FlowJo, LLC).

### 2.5 RNA-sequencing and data analysis

Maxillary periodontal tissues were collected for RNA extraction (from the same batch as that used for RT-qPCR). For each tissue type, RNA samples from the ligated side and the contralateral control side were subjected to RNA sequencing with n = 3 biological replicates per group. Libraries were prepared from total RNA, and mRNA was enriched by poly(A) selection to remove rRNA. Sequencing was performed on an Illumina NovaSeq 6000 platform with 2 × 150 bp paired-end reads. Raw reads were processed with Trimmomatic (v0.39) to remove adapters and low-quality sequences, and read quality was assessed using FastQC (v0.11.9). Clean reads were aligned to the mouse reference genome (GRCm39) using HISAT2 (v2.2.1). Transcript assembly and abundance estimation were performed with StringTie (v2.1.7) based on GRCm39 gene annotations. Differential expression analysis was conducted in R using DESeq2 (v1.32.0). Differential expression genes (DEGs) were defined using adjusted P < 0.05 and FDR<0.1, together with a fold-change cutoff of |log2(Fold Change) |≥1 (equivalent to FC ≥ 2). Volcano plots and heatmaps were generated in R, and Gene set enrichment analysis (GSEA) was performed in R using a preranked approach.

### 2.6 Cell culture

Primary BMSCs were isolated from male C57BL/6 diet-induced obese (DIO) mice. BMSCs were flushed from long bones and resuspended in αMEM medium with 10 % fetal bovine serum and 1 % penicillin-streptomycin mixture. The RAW264.7 macrophage cell line was obtained from the American Type Culture Collection (ATCC, USA) and cultured in high-glucose DMEM supplemented with palmitic acid (100–250 μM, PA; P5585, Sigma). For the indicated groups, LPS (100 ng/ml) or semaglutide (100 nM) was added to the culture medium for *in vitro* experiments [30].

### 2.7 FAO level analysis

FAO enzyme activities were quantitatively assessed using fatty acid oxidation detection reagent (5 μM, #FDV-0033, Funakoshi, Japan). Cells were incubated with HBS containing the dissolved FAO Blue at 37 °C for 30 min to allow for adequate staining. Following incubation, cells were stained with SYTO Green nuclear dye (1:1500, #KFS147, Beijing Baiaolaibo Technology, China). To assess FAO activity, the cells were visualized under a fluorescence microscope (BX53, OLYMPUS, Japan).

### 2.8 ALP staining and quantitation

ALP staining was performed on BMSCs with commercial kits (P0322M, Beyotime, Shanghai, China) according to the manufacturer's instructions. A semiquantitative analysis of the ALP activity of BMSCs was performed by using the ALP assay kit (P0321S, Beyotime, Shanghai, China). The total protein was measured using a bicinchoninic acid (BCA) assay kit (A045-4-2, Nanjing Jiancheng Bioengineering Institute, China). Metformin (100-1000 μm, Abcam, #ab120847) was added in the indicated experiments.

### 2.9 ARS staining

After culturing for 14 days, ARS staining and quantitative analysis were performed on BMSCs with commercial kits (C0140, Beyotime, Shanghai, China) according to the manufacturer's instructions.

### 2.10 Western blot

BMSCs were collected with RIPA buffer and then centrifuged at 12,000 rpm at 4 °C to obtain total protein. Western blot analysis was performed as previously described with AMPK (1:1000, #2532, Cell Signaling Technology., USA), p-AMPK (Thr172) (1:1000, #2531, Cell Signaling Technology., USA), CD36 (1:1000, #74002, Cell Signaling Technology., USA), CPT1A (1:1000, Abcam, #ab128568), RUNX2 (1:1000, #12556, Cell Signaling Technology., USA) and GAPDH (1:5000, D16H11, Cell Signaling Tech Inc., USA). For the indicated groups, compound C (10 μm, Abcam, #ab120843) or AICAR (2.5 mM, Abcam, #ab120358) was added to the culture medium for *in vitro* experiments.

### 2.11 Fabrication and characterization of the metformin@MPDA

MPDA was synthesized according to a reported method [31]: 0.36 g F127 and 0.36 g TMB were dissolved in H_2_O (65 mL)/ethanol (60 mL) and stirred for 30 min, followed by sequential addition of Tris (18 mg/mL, 5 mL) and dopamine (12 mg/mL, 5 mL) aqueous solutions; the mixture was stirred at room temperature for 24 h. The particles were collected by centrifugation (13,000 rpm, 10 min) and the templates (F127 and TMB) were removed by three sonication cycles (ethanol sonication for 30 min, centrifugation, then sonication in ethanol/acetone = 2/1, v/v); the final MPDA was suspended in ethanol. For metformin loading, various amounts of Met in PBS were added to MPDA dispersions and stirred at room temperature for 48 h, then centrifuged (13,000 rpm, 10 min) to remove unloaded Met and washed with PBS [32]. The Met@MPDA was characterized by a UV-vis spectrometer (Shimadzu, UV-1650PC, Kyoto, Japan), while the drug-loading capacity (DL%) andentrapment efficiency (EE%) were determined by HPLC (Agilent, Technologies 1260 Infinity, Santa Clara, USA). The size of MPDA and Met@MPDA were measured by dynamic light scattering analysis (Malvern Nano-ZS90, Malvern, UK). Morphology of MPDA and Met@MPDA was characterized by TEM (FEI, Tecnai G2 Spirit 120 KV, Hillsboro, USA). For cellular uptake assay, Cy5 modified MPDA-PEG nanoparticles were prepared by mixing 20 mg of MPDA-PEG nanoparticles with 5 mg of amine-terminated Cy5 in dimethyl sulfoxide (DMSO)/PBS (v/v, 1/99) solution by magnetic stirring for 24 h in dark. GelMA-GM60 was purchased from EFL (Engineering for Life, Suzhou, China). Following the manufacturer's instructions, the photoinitiator (0.25% w/v) was dissolved in deionized water, and GM60 was blended with the photoinitiator. Then, 10% (w/v) GelMA hydrogels were prepared by dissolving GelMA in the photoinitiator solution, maintaining it at 60–70 °C for 20–30 min, cooling to room temperature, and sterilizing by filtration through a 0.22 μm membrane. For *in vivo* injection, Met@MPDA was mixed with GelMA to prepare the formulation, which was subsequently crosslinked in situ by local injection followed by photo-crosslinking under 405 nm light for 10–30 s to achieve gelation. Evaluate the performance of Met@MPDA and GelMA using SEM, AFM, and tensile–compression tests.

For the degradation curve, the hydrogel was co-incubated with type II collagenase (0.5 U/mL) at 37 °C; the samples were weighed every day to record mass changes, and the weight loss/degradation rate was calculated accordingly. The cumulative release of met from Met@MPDA-Gel nanoparticles was evaluated under simulated periodontitis (pH 6.8) conditions. At scheduled intervals, the released met was quantified by UV–vis spectroscopy (using a met calibration curve).

### 2.12 Immunofluorescence staining

After washing with PBS, cells underwent fixation using 4 % paraformaldehyde, followed by permeabilization using a 0.1 % Triton X-100 solution for 15 min. Subsequently, they were treated with 5 % bovine serum albumin for 1 h to prevent nonspecific binding. Thereafter, cells were incubated with antibodies against OCN (AF6297, Beyotime, China) and F-actin (TRITC-phalloidin, 40736ES75, Yeasen, China) at 4 °C overnight, followed by fluorescent secondary antibodies for 2 h at room temperature as previously described. Cell nuclei were identified with DAPI (C1006, Beyotime, China). Fluorescent labeling was analyzed using a fluorescent upright microscope (BX51, Olympus). Fluorescence intensities were evaluated using ImageJ software.

### 2.13 Statistical analysis

Data were presented as mean ± standard error of the Mean (SEM) from at least three biological replicates of experiments. Statistical comparison was performed using Student's t-test, one-way ANOVA, or three-way ANOVA with a Tukey post-hoc analysis. Significance levels are as follows: *∗P < 0.05, ∗∗P < 0.01, ∗∗∗P < 0.001, ∗∗∗∗P < 0.0001*; ns indicates non-significant. All statistical analyses were performed using either GraphPad Prism 9.0.

## 3 Results

### 3.1 Obesity exacerbates periodontitis-associated alveolar bone loss, while semaglutide attenuates this effect

**Fig. 1:**
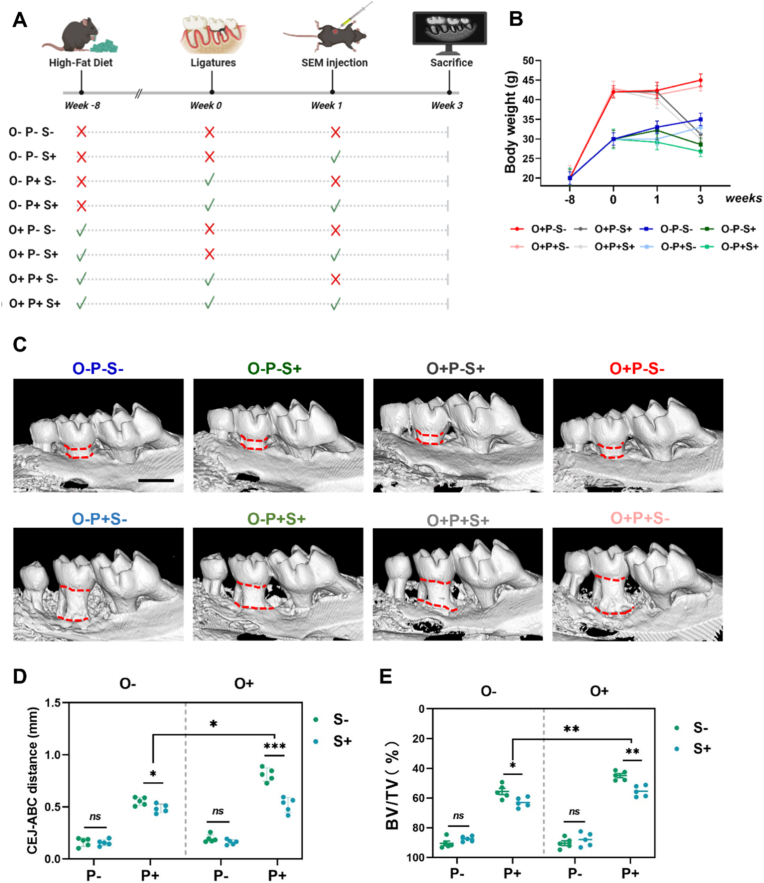
**Semaglutide mitigates inflammation-driven alveolar bone loss in an obesity–periodontitis mouse model. (A)** Experimental design and group allocation. Mice were fed a high-fat diet (obesity, O+) or control diet (O−). Periodontitis (P+) was induced by ligature placement at week 0 (sham, P−). Semaglutide (S+) or vehicle (S−) was administered by injection starting at week 1. Animals were sacrificed at week 3. **(B)** Body weight changes from baseline (week −8) to week 3 across groups. **(C)** Representative 3D micro-CT reconstructions of alveolar bone in each group; red dashed lines indicate the region used to assess alveolar bone loss. Scale bar = 1 mm. **(D**–**E)** Quantification of alveolar bone loss as the CEJ–ABC distance (mm) and BV/TV (%) in O− and O+ mice stratified by P and S status. Data are shown as individual values with mean ± SEM (n = 5). Statistical significance is indicated as *ns, not significant; ∗P<0.05, ∗∗P<0.01, ∗∗∗P<0.001.* (For interpretation of the references to colour in this figure legend, the reader is referred to the Web version of this article.)

First, we compared the effects of obesity, periodontitis, and semaglutide treatment on inflammation-associated alveolar bone loss. Mice were fed a high-fat diet for 8 weeks to induce obesity (O). Periodontitis (P) was induced by ligature placement for 1 week, after which semaglutide (S) injections were initiated and continued for 2 weeks (Fig. 1A). Semaglutide reduced body weight, and a similar weight-loss effect was also observed in normal-weight mice following semaglutide administration (Fig. 1B). Further micro-CT analyses revealed that periodontitis was the primary driver of alveolar bone loss, and that inflammation induced greater alveolar bone loss in obese mice (Fig. 1C). Quantitative analyses showed that semaglutide partially alleviated periodontal bone loss in obese mice, as evidenced by reduced CEJ–ABC and increased BV/TV compared with untreated obese controls (Fig. 1D and E). Collectively, these findings indicate that obesity exacerbates periodontitis-associated alveolar bone loss. Semaglutide mitigates inflammation-driven bone loss, particularly in the obese mice.

To clarify inflammation-driven alveolar bone remodeling in obese patients treated with injectable semaglutide, we retained five groups for subsequent analyses: O−P−S+, O−P + S−, O−P + S+, O + P + S−, and O + P + S+. The excluded groups (O−P + S+, O + P−S−, and O + P−S+) served only as non-core comparisons because they lacked sufficient periodontitis context or did not provide adequate discriminative contrast between obesity–inflammation and drug-delivery effects.

### 3.2 Obesity suppresses local Inflammation–Driven alveolar bone remodeling, and semaglutide further attenuates it: reduced osteoclastogenesis with limited bone formation

**Fig. 2:**
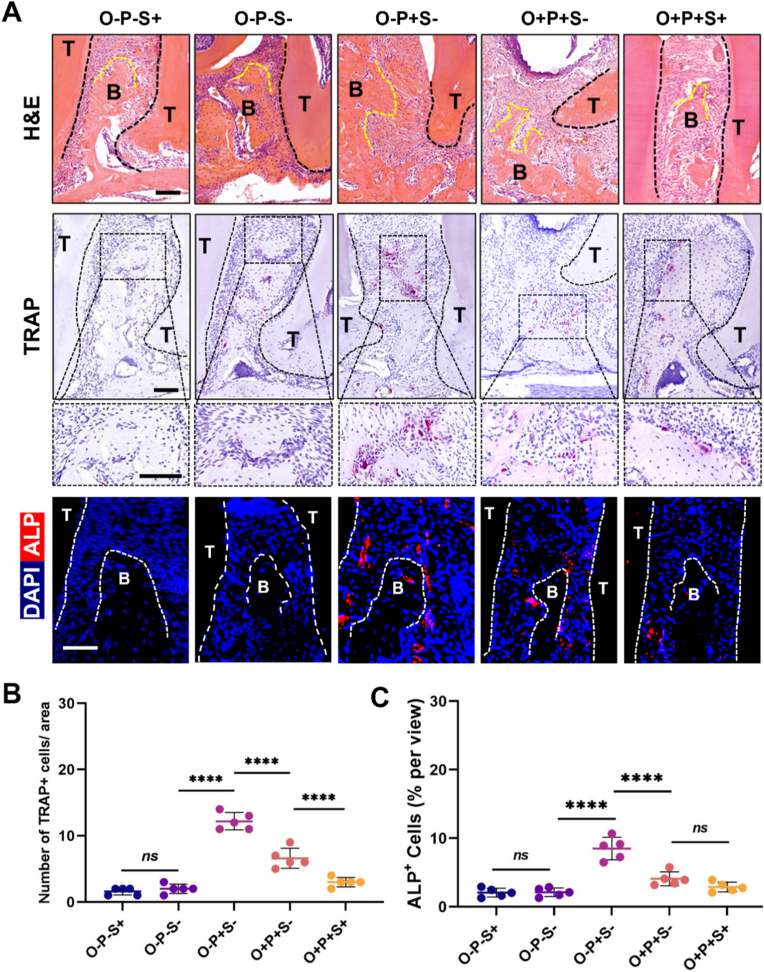
**Obesity Dampens Inflammation-Driven Bone Remodeling, While Semaglutide Partially Restores Alveolar Bone via Osteoclast Suppression. (A)** Representative images of alveolar bone sections from the indicated groups (O−P−S+, O−P−S−, O−P + S−, O + P + S−, O + P + S+). H&E (scale bar = 100 μm), TRAP (scale bar = 100 μm), and ALP staining (scale bar = 100 μm) are shown. Dashed outlines indicate the alveolar bone region; B, bone; T, tooth. **(B)** Quantification of TRAP^+^ cells per area. **(C)** Quantification of ALP^+^ cells (% per view). Data are shown as individual values with mean ± SEM (n = 5). Statistical significance is indicated as *ns, not significant; ∗∗∗∗P<0.0001.*

We further evaluated osteogenic and osteoclastic activities in periodontitis. H&E staining showed that in the periodontitis group (P+), trabecular architecture and bone surface morphology were markedly altered. In the inflammatory microenvironment, bone remodeling activity was enhanced: TRAP staining indicated an increase in osteoclast numbers, and ALP immunofluorescence showed elevated osteogenesis-associated signals (Fig. 2A). Quantitative analyses showed higher TRAP^+^ cell numbers and ALP^+^ cell proportions in the O−P+S− group than in the O−P−S− group, indicating active bone remodeling (Fig. 2B and C). Against this background, obesity (O+) blunted local inflammation–driven bone remodeling, with fewer TRAP^+^ cells and a lower proportion of ALP^+^ cells than the O-P + S- group. Moreover, in the O + P+S+ group, despite reduced TRAP^+^ cell numbers, ALP^+^ cell activity remained low.

The above findings indicate that, under inflammatory conditions, obesity may dampen bone remodeling activity. In the O + P+S+ group, alveolar bone showed partial recovery, likely due to reduced osteoclast levels; however, osteogenic potential remains insufficient in this context.

### 3.3 Obesity increases M1 macrophage infiltration in inflamed alveolar bone, while semaglutide reduces local M1 macrophages and promotes pro-reparative M2 macrophages

**Fig. 3:**
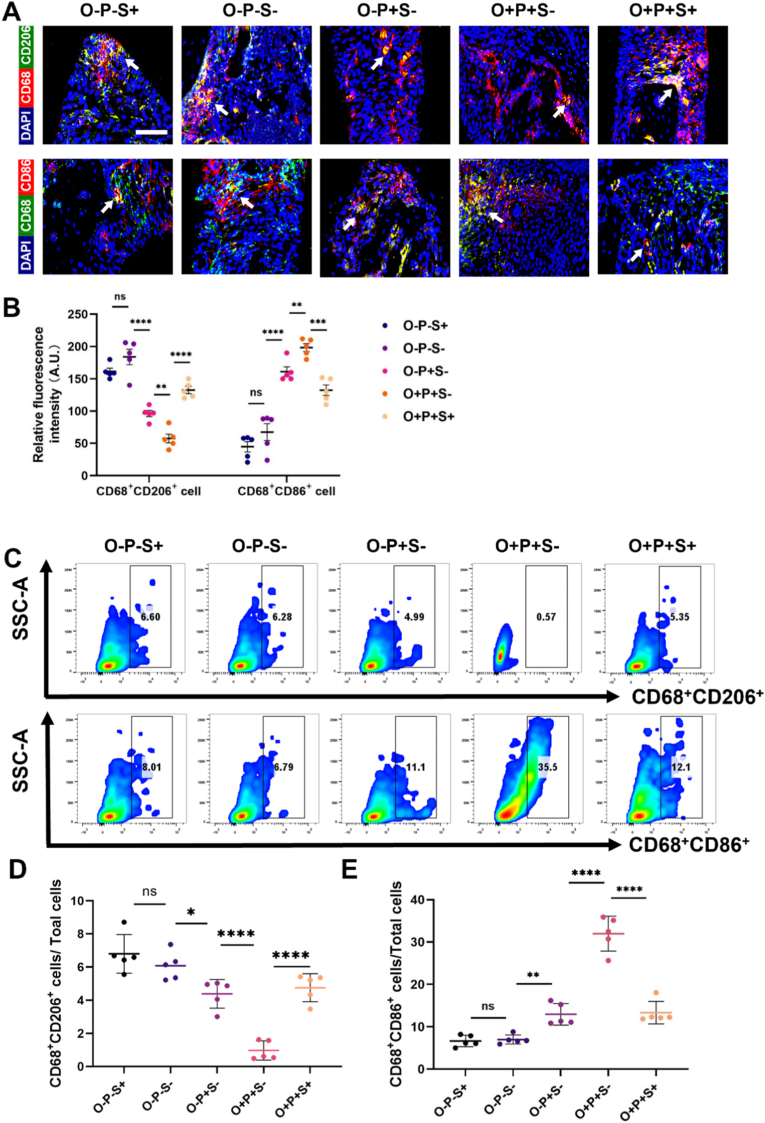
**Semaglutide influences macrophage polarization within the inflamed alveolar bone microenvironment under obese conditions. (A)** Representative immunofluorescence images showing CD68^+^CD206^+^ or CD68^+^CD86^+^ cells in inflamed alveolar bone across experimental groups. Arrows indicate representative double-positive cells. Scale bar = 100 μm. **(B)** Quantification of relative fluorescence intensity for CD68^+^CD206^+^ and CD68^+^CD86^+^ signals among groups. **(C)** Representative flow-cytometry gating plots for CD68^+^CD206^+^ and CD68^+^CD86^+^ macrophage populations in each group. **(D&E)** Flow-cytometry quantification of CD68^+^CD206^+^ and CD68^+^CD86^+^ cells expressed as a percentage of total cells. Data are shown as mean ± SEM (n = 5); significance is indicated as *ns, not significant; ∗P < 0.05, ∗∗P < 0.01, ∗∗∗P < 0.001, ∗∗∗∗P < 0.0001.*

To further clarify whether obesity and semaglutide affect osteogenesis by reshaping the local inflammatory microenvironment, we evaluated macrophage polarization in inflamed alveolar bone. Immunofluorescence staining showed distinct changes in the spatial distribution and co-localization of CD68 with CD86 (M1 type) or CD206 (M2 type) across groups (Fig. 3A and B), which were further confirmed by flow cytometry (Fig. 3C). Quantitative analyses showed that, compared with the O−P+S− group, obesity significantly reduced CD68^+^CD206^+^ (M2) macrophage infiltration in inflamed alveolar bone in the O + P+S− group, whereas semaglutide treatment markedly restored M2 macrophage enrichment (Fig. 3B and D). In parallel, obesity increased M1 macrophage accumulation, while semaglutide significantly decreased the proportion of CD68^+^CD86^+^ pro-inflammatory macrophages relative to obese inflamed controls (Fig. 3B and E).

These findings collectively indicate that obesity may alter the local immune environment towards an M1-dominant, pro-inflammatory state. In contrast, semaglutide seems to partially mitigate this alteration by reducing M1 macrophage accumulation and promoting M2-associated reparative polarization.

### 3.4 Semaglutide reprograms inflammatory and bone-remodeling gene expression by reducing AMPK-driven FAO in obesity-associated periodontitis

**Fig. 4:**
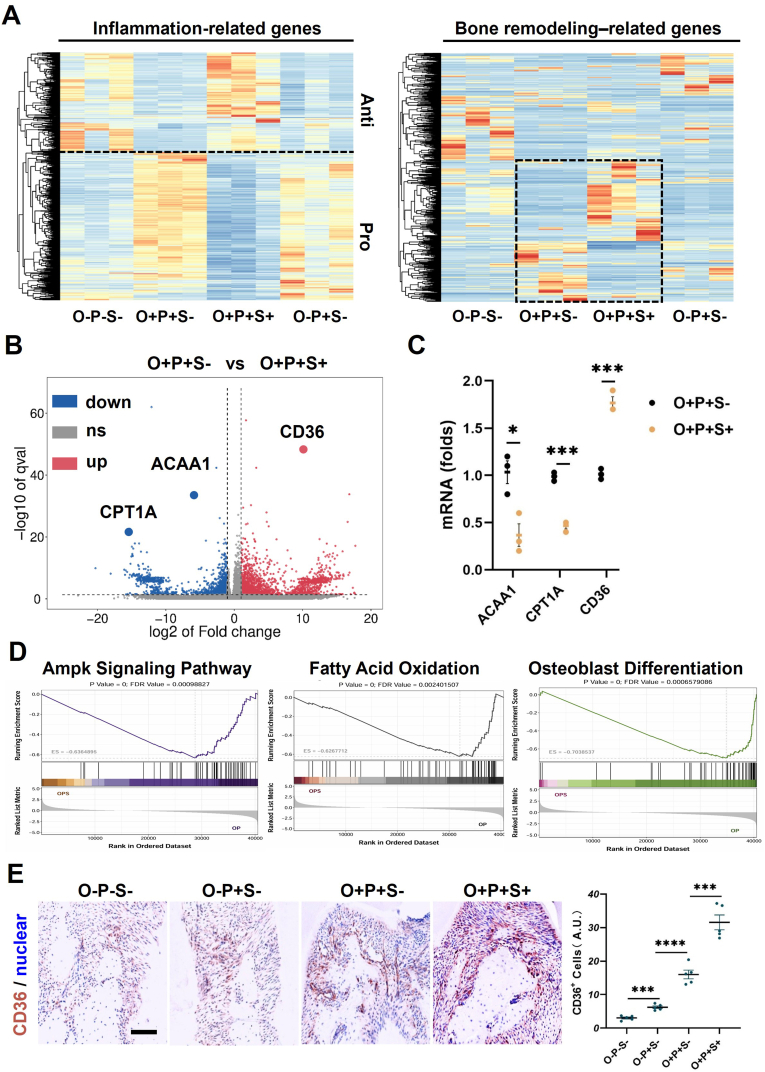
**Semaglutide Modulates AMPK-Driven FAO in Obesity-Inflamed Alveolar Bone. (A)** Heatmaps showing the expression patterns of inflammation-related genes and bone remodeling–related genes across the indicated groups. **(B)** Volcano plot of differentially expressed genes in O + P+S− vs O + P + S+. Significantly upregulated genes are shown in red and downregulated genes in blue. **(C)** qRT–PCR validation of ACAA1, CPT1A, and CD36 mRNA levels in O + P+S− and O + P + S+. Data are presented as fold change. **(D)** GSEA plots showing enrichment of the AMPK signaling pathway, FAO, and osteoblast differentiation signatures between O + P+S− and O + P + S+. **(E)** Representative immunohistochemical staining of CD36 in periodontal tissues from the indicated groups, with quantification of CD36^+^ cells. Scale bar = 100 μm. Data are shown as mean ± SEM (n = 5). Statistical significance: *∗P < 0.05, ∗∗∗P < 0.001, ∗∗∗∗P < 0.0001.* (For interpretation of the references to colour in this figure legend, the reader is referred to the Web version of this article.)

From the transcriptomic perspective, heatmaps showed clear separation among groups in the expression profiles of inflammation-related and bone remodeling–related genes (Fig. 4A), indicating that obesity accompanied by inflammatory challenge reshapes the local molecular microenvironment. Comparative analysis of the O + P+S− and O + P+S+ groups highlighted ACAA1, CPT1A, and CD36 as key differentially expressed genes (Fig. 4B). Lipid-metabolism–associated genes ACAA1 and CPT1A were significantly downregulated, whereas the fatty-acid transporter CD36 was upregulated. And these transcriptomic findings were further corroborated by tissue rt-qPCR analysis (Fig. 4C). GSEA showed that, compared with O + P + S−, the O + P+S+ group exhibited a concerted reduction in the enrichment of the AMPK signaling pathway and fatty acid oxidation, together with diminished enrichment of the osteoblast differentiation gene set (Fig. 4D). These results collectively indicate that semaglutide may influence osteogenic differentiation by modulating energy metabolism. Histologically, CD36 immunostaining revealed an increased number of CD36^+^ cells in the O+P+S− group; this increase was further augmented after semaglutide treatment, as supported by quantitative analysis (Fig. 4E).

Collectively, these data suggest that, in obesity-associated periodontitis, semaglutide may downregulate the AMPK-driven FAO pathway, thereby inducing local impairment of osteogenic differentiation.

### 3.5 Semaglutide impairs obesity-derived BMSC osteogenic differentiation by suppressing AMPK-driven fatty acid oxidation

**Fig. 5:**
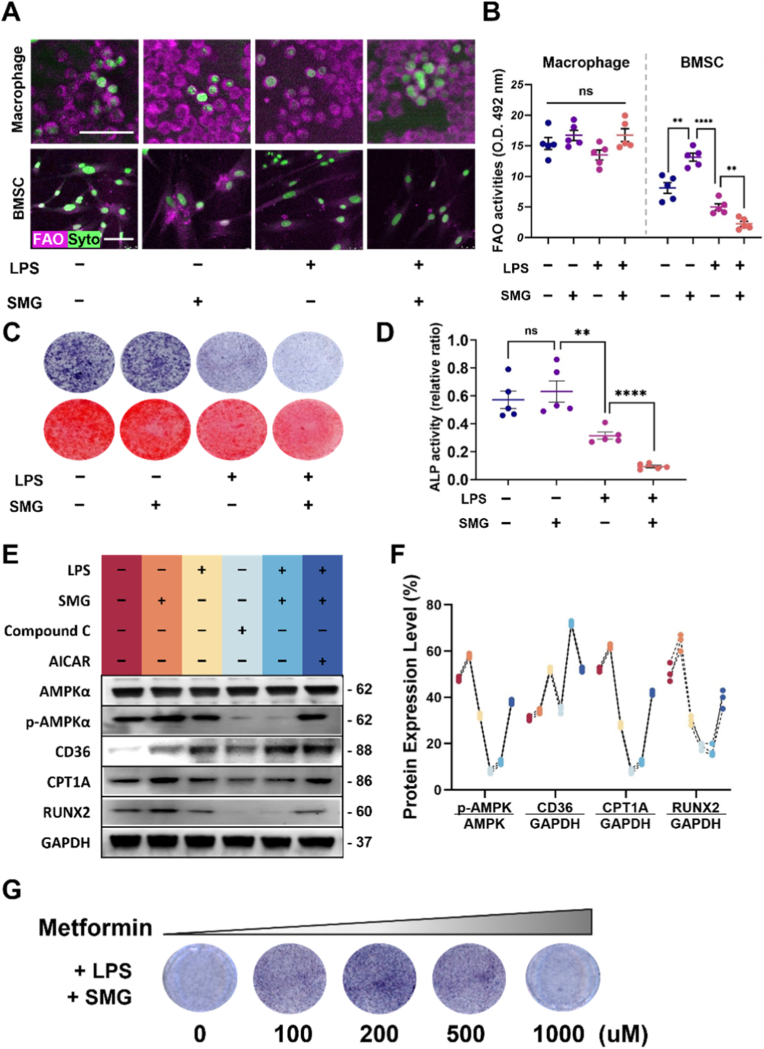
**Semaglutide attenuates BMSC osteogenic differentiation, which could be rescued by AMPK activation. (A)** Representative images of FAO and cell nucleus staining in macrophages and BMSCs treated with LPS and/or SMG as indicated. Scale bar = 50 μm. **(B)** Quantification of FAO activities in macrophages and BMSCs under the indicated treatments. **(C)** Representative images of ALP staining (upper) and Alizarin Red S staining (lower) of BMSCs following treatment with LPS and/or SMG. **(D)** Quantification of ALP activity in BMSCs across groups. **(E)** Western blot analysis of AMPK signaling, FAO, and osteogenic markers in BMSCs under various conditions. **(F)** Densitometric quantification of the western blots normalized to total AMPKα or GAPDH as indicated. **(G)** Representative ALP staining of BMSCs cultured under LPS + SMG conditions with increasing concentrations of metformin (0–1000 μM). Data are presented as mean ± SEM (n = 5). Statistical significance is indicated in the graphs (*ns, not significant; ∗∗P < 0.01; ∗∗∗∗P < 0.0001*). (For interpretation of the references to colour in this figure legend, the reader is referred to the Web version of this article.)

*In vitro*, obesity-derived BMSCs and macrophages under obese culture conditions exhibited markedly different energy-metabolic responses across conditions (Fig. S1). Specifically, neither LPS nor semaglutide (SMG) significantly affected FAO in macrophages. In contrast, BMSCs were more responsive: SMG modestly increased FAO, whereas LPS reduced it. Notably, combined treatment with LPS and SMG produced a pronounced suppression of FAO in BMSCs (Fig. 5A and B). As shown in Fig. S2, the expression levels of FAO-related proteins (CPT1A and ACADL) exhibited a similar trend across different conditions in both macrophages and BMSCs. Collectively, these data indicate that SMG can regulate FAO-related signaling under inflammatory conditions, supporting the mechanistic interpretation of our findings. Regarding osteogenic function, SMG had no significant effect on BMSC osteogenic differentiation under non-inflammatory conditions; however, under inflammatory stimulation, SMG appeared to further restrict osteogenic differentiation. ALP staining and activity assays showed a significant reduction in ALP activity in the LPS + SMG group (Fig. 5C and D). Mechanistically, the western blotting results showed that AMPK activation (increased p-AMPK/AMPK) occurred after 1.5 h (Fig. S3). SMG further activated AMPK and upregulated CPT1A and CD36, concomitantly increasing RUNX2.

Mechanistically, Western blotting showed that SMG activated AMPK (increased p-AMPK/AMPK) and upregulated CPT1A and CD36, along with increased RUNX2. Inflammatory stimulation reduced AMPK signaling and lowered CPT1A and RUNX2. Notably, the LPS + SMG group showed a pattern similar to AMPK inhibition by Compound C, with suppressed osteogenic markers, whereas the AMPK agonist AICAR partially restored osteogenic differentiation of BMSCs under these conditions (Fig. 5E and F). As an AMPK agonist, metformin was further evaluated at different concentrations for its effects on BMSC osteogenic differentiation. It was found that metformin at 200 μM significantly promoted osteogenic differentiation in BMSCs.

### 3.6 Development of a Met@MPDA Microsphere–Based sustained-release system

**Fig. 6:**
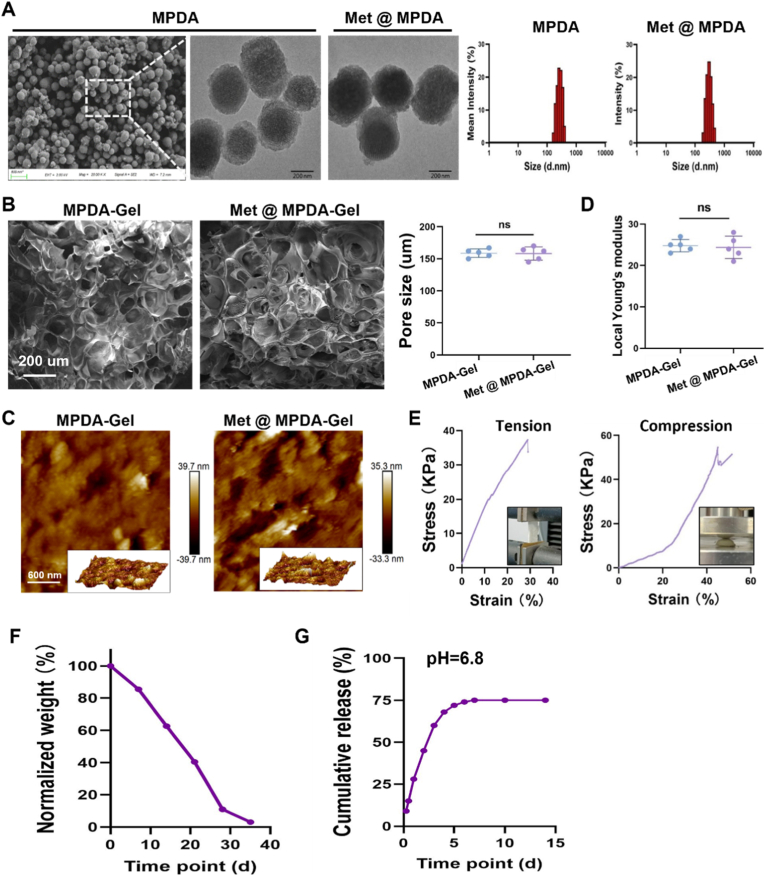
**Construction and characterization of Met@MPDA nanoparticles and Met@MPDA-Gel hydrogel. (A)** Representative TEM images and DLS size distributions of MPDA nanoparticles before and after metformin loading (Met@MPDA). **(B)** SEM images of MPDA-Gel and Met@MPDA-Gel, with quantification of pore size. Scale bar = 200 μm. **(C)** AFM topography images of MPDA-Gel and Met@MPDA-Gel. Scale bar = 600 nm. **(D)** Local Young's modulus of MPDA-Gel and Met@MPDA-Gel. **(E)** Representative tensile and compressive stress–strain curves of the hydrogel. **(F)***In vitro* degradation profile of the hydrogel presented as normalized weight over time. **(G)** Cumulative metformin release from Met@MPDA-Gel at pH 6.8 over time. Data are presented as mean ± SEM; *ns, not significant.*

To demonstrate the effects of an AMPK agonist, we established a sustained-release system for metformin by loading metformin into MPDA and incorporating it into the hydrogel matrix. MPDA nanoparticles exhibited a uniform spherical morphology. After metformin loading (Met@MPDA), the particle morphology remained unchanged, and the size distribution indicated no obvious shift in particle size (Fig. 6A). Incorporation of the nanoparticles into the hydrogel produced an interconnected porous architecture in both MPDA-Gel and Met@MPDA-Gel, with comparable pore sizes and no statistically significant difference between groups (Fig. 6B). AFM imaging further confirmed similar surface microtopography for the two hydrogels (Fig. 6C). Consistently, the local Young's modulus showed no significant change after introducing Met@MPDA (Fig. 6D), and the tensile and compressive stress–strain curves demonstrated good deformability and mechanical stability of the hydrogels (Fig. 6E). The hydrogel underwent time-dependent degradation, as reflected by the gradual decrease in normalized weight over time (Fig. 6F). Under mildly acidic conditions (pH 6.8), metformin release displayed an initial rapid phase followed by a plateau, reaching a stable cumulative release thereafter (Fig. 6G).

### 3.7 Validation of Met@MPDA–mediated restoration of osteogenesis in semaglutide-treated obese periodontitis mice

**Fig. 7:**
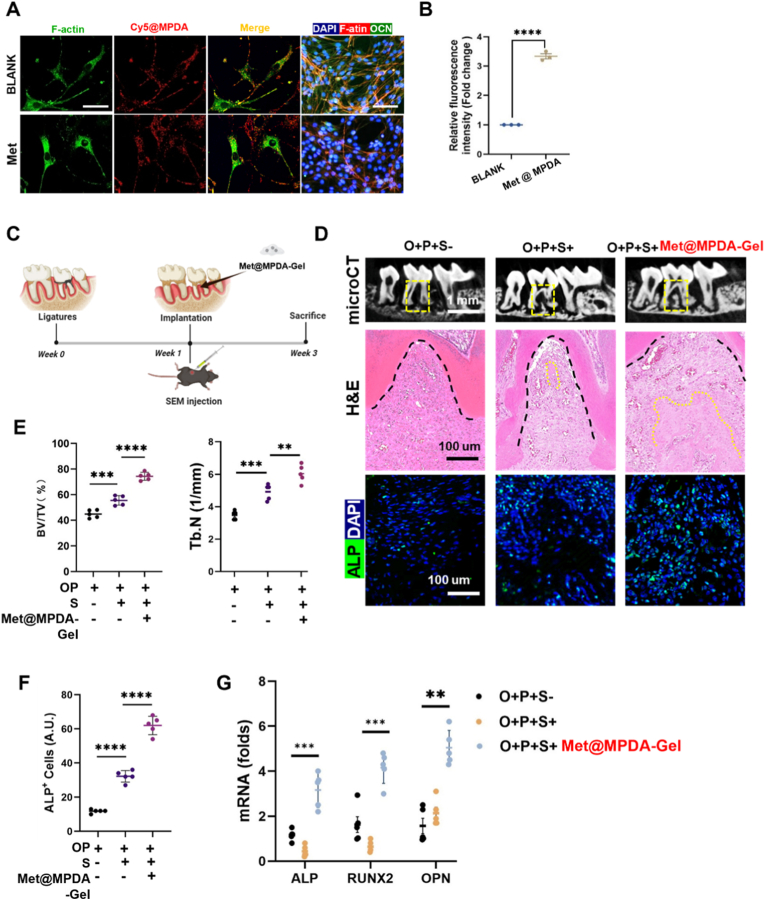
***In vitro* and *in vivo* validation of Met@MPDA–mediated restoration of osteogenesis. (A)** Confocal laser scanning microscopy images of BMSCs treated with Cy5@MPDA for 4 h. Scale bar = 50 μm. **(B)** Quantification of intracellular fluorescence intensity in the BLANK and Met@MPDA groups. **(C)** Schematic timeline of the *in vivo* study in obese mice with ligature-induced periodontitis. **(D)** Representative micro-CT images, H&E staining, and ALP immunofluorescence staining of the defect region under the indicated treatments (O + P + S−, O + P + S+, and O + P + S + Met@MPDA-Gel). Yellow boxes indicate the corresponding ROI; dashed outlines delineate the regenerative area. Scale bars as indicated. **(E)** Quantification of alveolar bone regeneration as the BV/TV (%) and Tb.N (1/mm) in the indicated groups. **(F)** Quantification of ALP^+^ cells in the defect region. **(G)** mRNA relative fold change of osteogenic genes across the different groups**.** Data are presented as mean ± SEM (n = 5); *∗∗P < 0.05, ∗∗∗P < 0.001, ∗∗∗∗P < 0.0001.* (For interpretation of the references to colour in this figure legend, the reader is referred to the Web version of this article.)

The biocompatibility of Met@MPDA was first verified by both *in vivo* and *in vitro* studies (Fig. S4). Cellular uptake analysis showed robust internalization of Met@MPDA by BMSCs, as evidenced by markedly stronger intracellular fluorescence signals compared with the blank group (Fig. 7A). Consistent with this, Met@MPDA treatment enhanced the osteogenic phenotype *in vitro*, with increased OCN-associated staining (Fig. 7A and B). To further evaluate its therapeutic potential *in vivo*, we established a ligature-induced periodontal bone loss model in obese mice. Semaglutide was administered systemically, while Met@MPDA-Gel was delivered locally to the periodontal site (Fig. 7C). Compared with the control groups, Met@MPDA-Gel treatment markedly enhanced regeneration-associated outcomes in the defect region, promoting the regeneration of well-organized tissue architecture with reduced inflammatory infiltration (Fig. 7D and E). Consistently, immunofluorescence revealed stronger and more broadly distributed ALP signals in the Met@MPDA-Gel group (Fig. 7D). Quantitative analyses further confirmed a significant increase in ALP^+^ cells and the expression of osteogenesis-related genes (ALP, RUNX2, OPN) in the Met@MPDA-Gel group compared with the O + P+S− and O + P+S+ groups. These results suggest that Met@MPDA effectively promotes early osteogenic activity and accelerates tissue repair (Fig. 7F and G).

## 4 Discussion

In this study, we demonstrate that obesity exacerbates alveolar bone loss associated with periodontitis, while semaglutide partially mitigates this degradation. Under conditions of obesity, the remodeling pattern becomes unfavorable for regeneration—bone resorption is somewhat restrained, yet bone formation is even more limited. Semaglutide further suppresses osteoclastogenesis but results in only modest improvements in bone formation. Consistent with its immunomodulatory effects, obesity increases M1 macrophage infiltration in inflamed alveolar bone, whereas semaglutide reduces local M1 macrophages and promotes a pro-reparative shift towards M2 polarization. However, we identify a mechanistic paradox: despite its anti-inflammatory and anti-resorptive actions, semaglutide is associated with suppression of the AMPK–FAO axis, reprogramming the expression of inflammatory and bone-remodeling genes, and directly limiting the osteogenic differentiation of BMSCs. To overcome this metabolic bottleneck, we developed a sustained-release Met@MPDA system and validated its efficacy in restoring osteogenesis in semaglutide-treated obese periodontitis models.

Our findings indicate that obesity exacerbates alveolar bone loss associated with periodontitis and creates a bone-remodeling environment inherently less favorable for regeneration. This phenotype likely results from the combined effects of systemic metaflammation, increased local immune activation, and obesity-related stromal cell dysfunction, collectively disrupting the coupling between bone resorption and formation [33]. Supporting our observations, a study on ligature-induced periodontitis in Wistar rats demonstrated greater palatal alveolar bone loss in diet-induced obese animals (high-fat diet) compared to non-obese controls, although the effect size was modest and appeared to be site-specific [34]. Clinical evidence further corroborates an inflammation-centered link between obesity and periodontal degradation: the periodontal inflamed surface area (PISA) has been shown to positively correlate with body mass index (BMI) in obese patients [35]. Mechanistic studies suggest that shared inflammatory pathways, such as increased cytokine signaling, are fundamental to the obesity–periodontitis association [36]. In addition to its role in inflammation, a growing body of evidence suggests that obesity may exacerbate alveolar bone resorption by disrupting bone metabolism [37]. Metabolic abnormalities, including insulin resistance and dysregulated adipokine secretion, can impair bone remodeling processes, thereby contributing to alveolar bone loss [38]. Collectively, these findings highlight that obesity functions not only as a catalyst for systemic metabolic dysfunction but also as a complex modifier of periodontal disease. It amplifies tissue degradation and bone loss through interconnected inflammatory, metabolic, and molecular pathways.

Emerging evidence indicates that semaglutide holds therapeutic promise in enhancing osteogenesis and modulating inflammation [39,40]. Its pro-osteogenic effects appear to be mediated, at least partially, through the regulation of the Wnt/β-catenin signaling pathway. A study demonstrated that semaglutide promotes bone formation by upregulating β-catenin expression and inhibiting RANKL signaling, thereby mitigating osteoporotic characteristics in an ovariectomized rat model [18]. This mechanism aligns conceptually with the bone-protective effects of semaphorin 3A, which inhibits RANKL-induced osteoclast differentiation while promoting osteoblast formation. Regarding inflammation control, semaglutide exhibits significant anti-inflammatory properties. Systematic reviews and meta-analyses have associated semaglutide treatment with notable reductions in C-reactive protein levels [41]. Furthermore, experimental studies suggest that semaglutide can reduce ovarian inflammation in a mouse model of polycystic ovary syndrome through the AMPK/SIRT1/NF-κB pathway [42]. In a model of acute lung injury induced by LPS, semaglutide significantly attenuated pulmonary damage and inflammatory responses by inhibiting HDAC5-mediated NF-κB signaling [16]. These findings collectively indicate that semaglutide may modulate the inflammatory trajectory within the alveolar bone microenvironment through partially overlapping signaling pathways. Overall, the expanding body of evidence supporting the roles of semaglutide in osteogenesis and inflammatory regulation enhances the understanding of its multi-system effects beyond metabolic disorders.

We hypothesize that insufficient activation of AMPK-driven FAO provides a mechanistic explanation for the limited effectiveness of semaglutide's anti-inflammatory and anti-resorptive actions in achieving robust alveolar bone regeneration. In obesity-associated periodontitis, semaglutide can modulate transcriptional programs governing inflammation and bone remodeling; however, it may simultaneously suppress AMPK-dependent FAO. Functionally, AMPK activation couples metabolic reprogramming to bone regeneration by promoting FAO and FAO-related lipid-metabolism signaling. Specifically, when AMPK is activated, it redirects energy utilization toward FAO through upregulation of FAO machinery and key regulators such as CPT1A (fatty-acid mitochondrial entry) and CD36 (fatty-acid uptake), thereby enhancing mitochondrial energetic capacity and supporting lipid handling under inflammatory stress. Because osteogenic differentiation of BMSCs is an energy-demanding process that depends on competent mitochondrial metabolism, attenuation of the AMPK–FAO axis is expected to limit osteogenic commitment and impair subsequent functional maturation [43,44]. Consistent with this cell type–specific model, semaglutide may alleviate inflammation in immune cells, while concurrently constraining BMSC metabolic adaptability, resulting in an “effective anti-inflammatory response but inadequate regenerative capacity” phenotype. Our data support this integrated framework: the concurrent modulation of FAO-related proteins and therapeutic benefit suggests that restoring AMPK-driven FAO activity is a key determinant of improved regenerative outcomes within the inflammatory osteogenic microenvironment, in agreement with published evidence that GLP-1RA can both promote osteogenic differentiation and attenuate inflammation [45].

To directly address the identified metabolic deficiency, we selected metformin as a prototypical AMPK agonist, known for its ability to enhance FAO, improve mitochondrial bioenergetics, and potentially offer additional anti-inflammatory effects [46]. Previous research has demonstrated that metformin, through AMPK activation, increases FAO rates and significantly enhances mitochondrial respiratory activity, membrane potential, and ATP production [47]. This, in turn, mitigates disturbances in glucose homeostasis induced by high-fat diets and reduces intracellular lipid accumulation. Furthermore, metformin has been shown to restore mitochondrial respiratory function by promoting mitochondrial fission and re-establishing mitochondrial quality control and turnover [48]. For delivery purposes, we utilized MPDA-based microspheres for localized administration. These microspheres possess a high drug-loading capacity and a sustained-release profile, which are expected to enhance effective local exposure while minimizing systemic distribution and associated adverse effects [49]. The mesoporous architecture of MPDA provides an excellent platform for efficient drug encapsulation and controlled release, demonstrating significant advantages in targeted drug delivery applications.

When interpreting these findings, several limitations should be acknowledged. First, the Met@MPDA platform requires rigorous characterization of its local retention, release kinetics, and batch-to-batch consistency, together with further assessment of biocompatibility and potential long-term metabolic consequences. Second, clinical translation may be influenced by differences between animal models and human disease, including variability in obesity severity and metabolic phenotypes, heterogeneity in periodontitis presentation and stage, dosing and treatment schedules, and the presence of comorbidities and concomitant medications—all of which may shape therapeutic responses and regenerative outcomes. These issues warrant validation in clinically relevant models and prospective studies.

## 5 Conclusion

Based on these findings, we propose a conceptual model in which obesity amplifies inflammation while impairing AMPK-driven FAO in BMSCs, thereby worsening bone loss and constraining bone formation. Semaglutide improves the local immune microenvironment and suppresses osteoclastogenesis; however, its concurrent inhibition of the AMPK-FAO axis in BMSCs limits osteogenic capacity and results in incomplete regenerative recovery. Local delivery of Met@MPDA may reactivate the AMPK–FAO pathway, thereby “refueling” osteogenesis and promoting alveolar bone regeneration. Collectively, these results suggest that for obesity-associated periodontitis, a dual-target therapeutic strategy that couples immunomodulation with metabolic reprogramming of BMSCs may be more effective and more reliable than monotherapy. Clinically, this supports the potential translation of Met@MPDA as a locally delivered, mechanism-based approach to improve periodontal regeneration in the context of obesity.

## CRediT authorship contribution statement

**Ting Jiang:** Conceptualization, Data curation, Resources, Writing – original draft, Writing – review & editing. **Tian-Hao Wan:** Data curation, Investigation, Project administration, Software, Validation. **Min-Jie Wang:** Conceptualization, Methodology, Software, Visualization. **Xue-Qin Zhu:** Data curation, Methodology, Resources, Validation. **Yu-Ran Jiang:** Investigation, Resources, Supervision, Validation, Visualization. **Feng Yang:** Formal analysis, Funding acquisition, Project administration, Supervision. **Zhi-Chen Ling:** Investigation, Resources, Validation. **Xin-Yi Tan:** Formal analysis, Methodology, Project administration, Visualization. **Jun Wang:** Resources, Visualization, Writing – review & editing. **Ning-Juan Ouyang:** Conceptualization, Data curation, Project administration, Writing – original draft.

## Declaration of competing interest

The authors declare that they have no known competing financial interests or personal relationships that could have appeared to influence the work reported in this paper.

## Data Availability

Data will be made available on request.
