# Supplementary material for: Met@MPDA rejuvenates BMSC energy metabolism to promote bone regeneration in semaglutide-treated obese periodontitis
Source: Mater Today Bio. 2026 May 14;38:103231. doi: 10.1016/j.mtbio.2026.103231 (PMC13197780; doi:10.1016/j.mtbio.2026.103231)
Supplement: Multimedia component 1 [file mmc1.docx]

**Supplement**


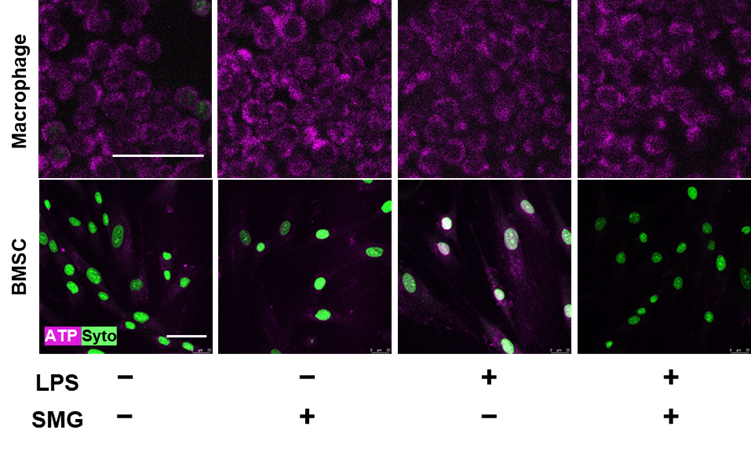
**Figure S1. ATP levels in macrophages and BMSCs across different conditions.** Representative immunofluorescence images showing ATP staining and.

**
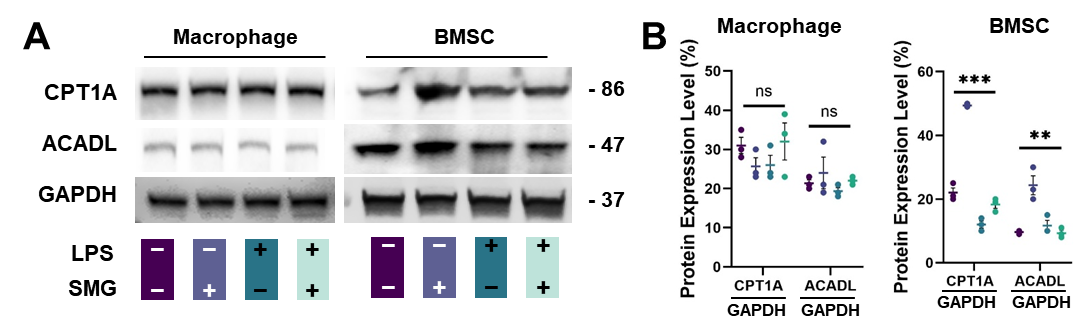
**

**Figure S2. The expression levels of FAO-related proteins in macrophages and BMSCs under different conditions. (A)** Representative western blot images of FAO-related proteins (CPT1A, ACADL) in macrophages and BMSCs treated with LPS and/or SMG as indicated. **(B)** Quantification of FAO-related proteins in macrophages and BMSCs under the indicated treatments. Data are shown as individual values with mean ± SEM (n=3). Statistical significance is indicated as *ns, not significant; **P<0.01, ***P<0.001.*


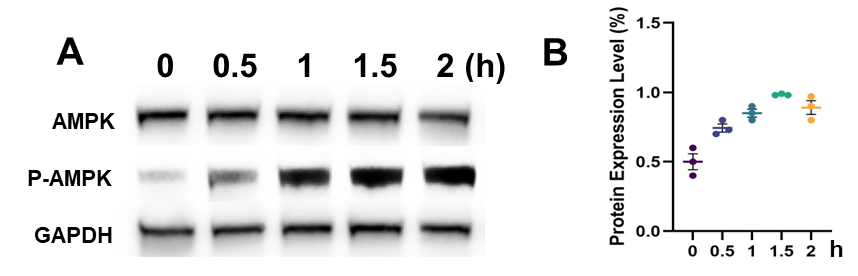
**Figure S3. Time-course analysis of early p-AMPK signaling under inflammatory conditions.** A representative Western blot showing p-AMPK at different time points, along with the corresponding quantitative analysis.


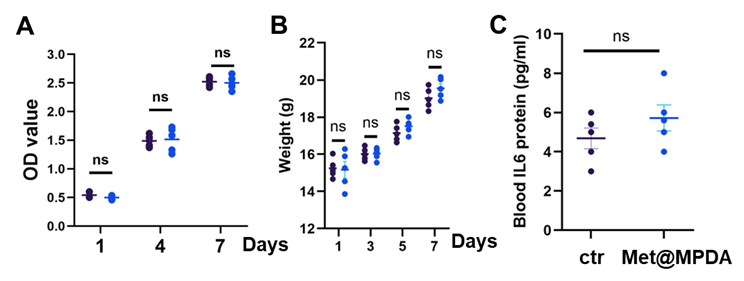


**Figure S4 Assessment of the biocompatibility of Met@MPDA. (A)** In vitro CCK-8 OD values of BMSCs. **(B)** Body weights of mice in different groups. **(C)** Serum IL-6 levels. Data are shown as individual values with mean ± SEM (n=5). Statistical significance is indicated as *ns, not significant.*
